# Supplementary material for: (-)-α-Pinene reduces quorum sensing and Campylobacter jejuni colonization in broiler chickens
Source: PLoS One. 2020 Apr 1;15(4):e0230423. doi: 10.1371/journal.pone.0230423 (PMC7112227; doi:10.1371/journal.pone.0230423)
Supplement: S1 Fig — Data are means ±standard deviation. Framed time points (i.e. 9 h) are those best suited for further evaluation of quorum sensing inhibition. (DOCX) [file pone.0230423.s001.docx]

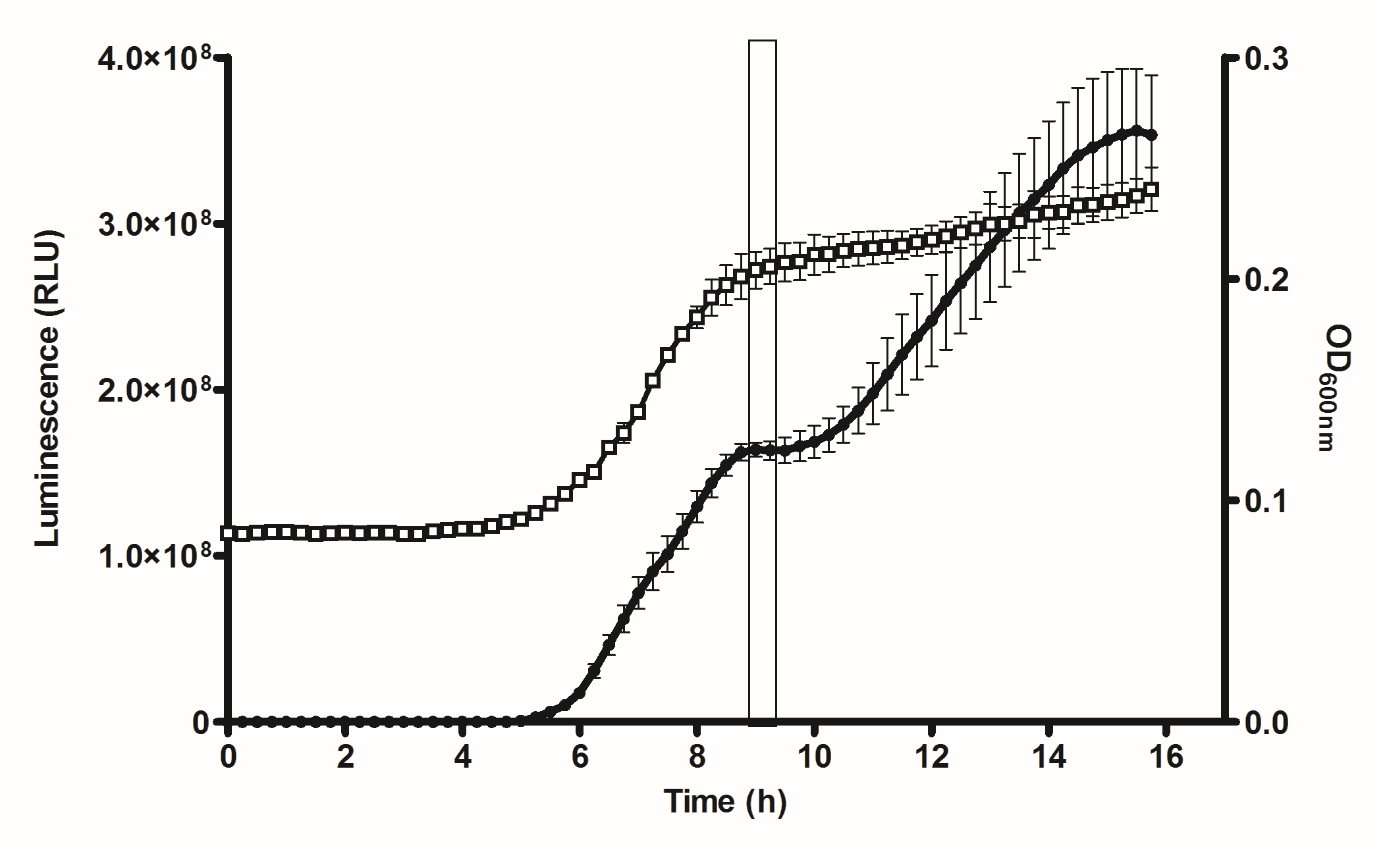


**Supplementary Figure S1.** Luminescence production (hollow squares) in relative luminescent units (RLU) and growth of *V. harveyi* BB170 (OD_600nm_; full circles) in AB medium supplemented with 20% of MHB. Presented are means with standard deviation. Framed are time points (i.e. 9 h) best suited for further evaluation calculations of quorum sensing inhibition.
