# Supplementary material for: (-)-α-Pinene reduces quorum sensing and Campylobacter jejuni colonization in broiler chickens
Source: PLoS One. 2020 Apr 1;15(4):e0230423. doi: 10.1371/journal.pone.0230423 (PMC7112227; doi:10.1371/journal.pone.0230423)
Supplement: S2 Fig — Data are means ±standard deviation for luminescent signal (relative luminescent units, RLU). Framed time point is that used in the calculation of quorum sensing inhibition. (DOCX) [file pone.0230423.s002.docx]

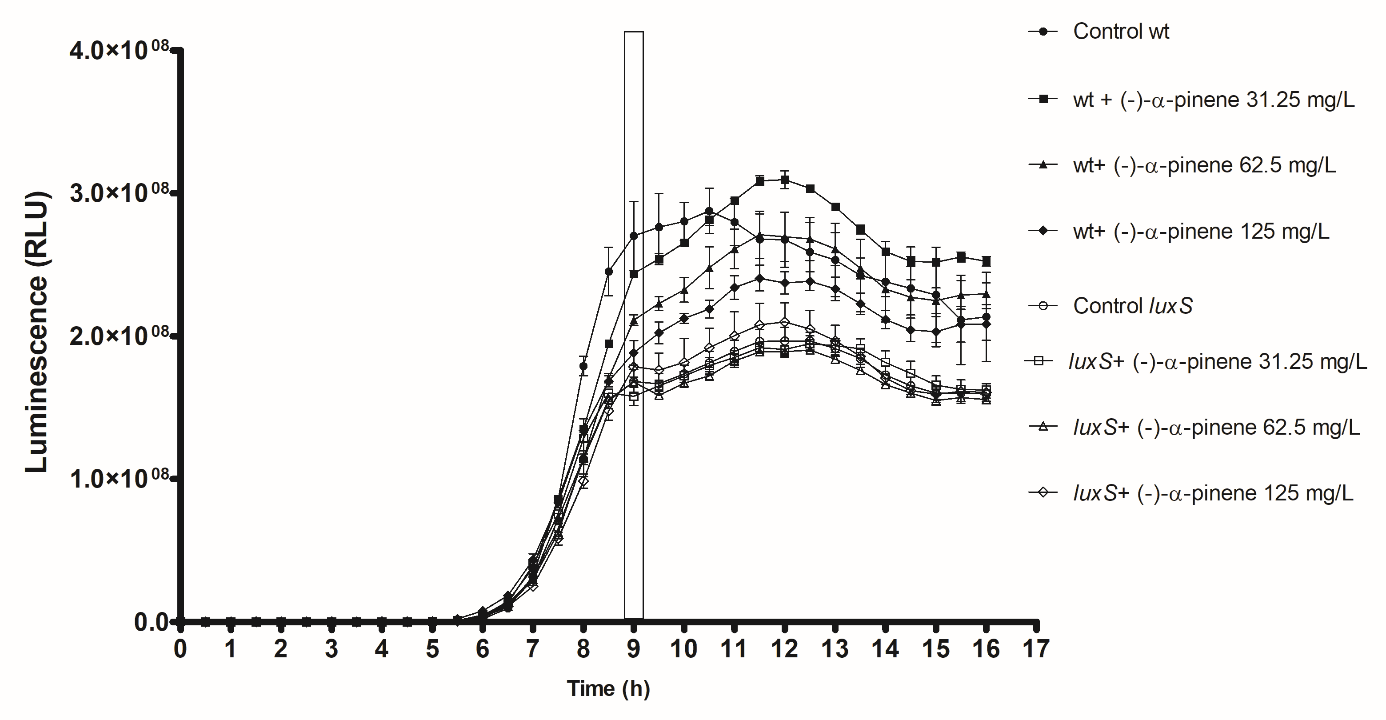


**Supplementary Figure S2**. Luminescence of *V. harveyi* BB170 produced after addition of cell free supernatants of *C. jejuni* wild type (wt control; full marks) and the Δ*luxS* mutant (*luxS*; hollow marks) without treatment (circles) and after treatment with 31.25 mg/L (square), 62.5 mg/L (triangle), and 125 mg/L (diamond) of (-)-α-pinene. Shown are means of luminescent signal in relative luminescent units (RLU) with standard deviation. Framed is the time point used in calculation of quorum sensing inhibition.
