# Supplementary material for: (-)-α-Pinene reduces quorum sensing and Campylobacter jejuni colonization in broiler chickens
Source: PLoS One. 2020 Apr 1;15(4):e0230423. doi: 10.1371/journal.pone.0230423 (PMC7112227; doi:10.1371/journal.pone.0230423)
Supplement: S1 Table — (DOCX) [file pone.0230423.s003.docx]

**Supplementary Table S1.** *Campylobacter jejuni* strains used in the study.

| **Strain code** | **Reported source** | **Reference** |
| --- | --- | --- |
| CB1:6 | Broiler | Luangtongkum et al., 2006 |
| CB1:14 | Broiler | Luangtongkum et al., 2006 |
| CB1:18 | Broiler | Luangtongkum et al., 2006 |
| CB2:6 | Broiler | Luangtongkum et al., 2006 |
| CB2:8 | Broiler | Luangtongkum et al., 2006 |
| CB2:11 | Broiler | Luangtongkum et al., 2006 |
| CB3:1 | Broiler | Luangtongkum et al., 2006 |
| CB3:5 | Broiler | Luangtongkum et al., 2006 |
| CB 4:21 | Broiler | Luangtongkum et al., 2006 |
| CB 4:22 | Broiler | Luangtongkum et al., 2006 |
| CB 6:8 | Broiler | Luangtongkum et al., 2006 |
| CB 6:9 | Broiler | Luangtongkum et al., 2006 |
| CB 6:26 | Broiler | Luangtongkum et al., 2006 |
| CB 7:15 | Broiler | Luangtongkum et al., 2006 |
| CB 7:21 | Broiler | Luangtongkum et al., 2006 |
| CB 8:14 | Broiler | Luangtongkum et al., 2006 |
| CB 8:15 | Broiler | Luangtongkum et al., 2006 |
| CT 1:1 | Turkey | Luangtongkum et al., 2006 |
| CT 1:9 | Turkey | Luangtongkum et al., 2006 |
| CT 2:2 | Turkey | Luangtongkum et al., 2006 |
| CT 3:5 | Turkey | Luangtongkum et al., 2006 |
| CT3:11 | Turkey | Luangtongkum et al., 2006 |
| CT3:19 | Turkey | Luangtongkum et al., 2006 |
| CT4:4 | Turkey | Luangtongkum et al., 2006 |
| CT4:14 | Turkey | Luangtongkum et al., 2006 |
| CT5:2 | Turkey | Luangtongkum et al., 2006 |
| CT5:8 | Turkey | Luangtongkum et al., 2006 |
| CT5:10 | Turkey | Luangtongkum et al., 2006 |
| CT5:12 | Turkey | Luangtongkum et al., 2006 |
| CT5:18 | Turkey | Luangtongkum et al., 2006 |
| CT 6:8 | Turkey | Luangtongkum et al., 2006 |
| CT 6:16 | Turkey | Luangtongkum et al., 2006 |
| CT 6:18 | Turkey | Luangtongkum et al., 2006 |
| CT 7:2 | Turkey | Luangtongkum et al., 2006 |
| CT 8:22 | Turkey | Luangtongkum et al., 2006 |
| CT 8: 28 | Turkey | Luangtongkum et al., 2006 |
| CT 8:29 | Turkey | Luangtongkum et al., 2006 |
| CT 9:14 | Turkey | Luangtongkum et al., 2006 |
| CT 9:21 | Turkey | Luangtongkum et al., 2006 |
| CT 10:18 | Turkey | Luangtongkum et al., 2006 |
| F6501 | Human fecal | Luangtongkum et al., 2006 |
| H2958 | Human fecal | Luangtongkum et al., 2006 |
| M63885 | Human fecal | Luangtongkum et al., 2006 |
| T59822 | Human fecal | Luangtongkum et al., 2006 |
| W14861 | Human fecal | Luangtongkum et al., 2006 |
| X60179 | Human fecal | Luangtongkum et al., 2006 |
| F15871 | Human fecal | Luangtongkum et al., 2006 |
| W11805 | Human fecal | Luangtongkum et al., 2006 |
| M402 | Human fecal | Luangtongkum et al., 2006 |
| W28752 | Human fecal | Luangtongkum et al., 2006 |
| M33323 | Human fecal | Luangtongkum et al., 2006 |
| W64861 | Human fecal | Luangtongkum et al., 2006 |
| M76297 | Human fecal | Luangtongkum et al., 2006 |
| E46972 | Human fecal | Luangtongkum et al., 2006 |
| M36292 | Human fecal | Luangtongkum et al., 2006 |
| X7199 | Human fecal | Luangtongkum et al., 2006 |
| 11168, reference strain | National Collection of Type Cultures | https://www.phe-culturecollections.org.uk/products/bacteria/ |
| 11168Δ*luxS*, insertional mutant in gene *luxS* |  | Bezek et al., 2016 |
