# Supplementary material for: (-)-α-Pinene reduces quorum sensing and Campylobacter jejuni colonization in broiler chickens
Source: PLoS One. 2020 Apr 1;15(4):e0230423. doi: 10.1371/journal.pone.0230423 (PMC7112227; doi:10.1371/journal.pone.0230423)
Supplement: S2 Table — (DOCX) [file pone.0230423.s004.docx]

**Supplementary Table S2.** Susceptibility of *Campylobacter jejuni* broiler and turkey strains to the range of tested antibiotics, as MIC values and corresponding sensitivity (S) or resistance (R).

| **Strain code** | **Antibiotic MIC (mg/L) and strain sensitivity (S/R)^a^** | | | | | | | | | | | | | | | | | |
| --- | --- | --- | --- | --- | --- | --- | --- | --- | --- | --- | --- | --- | --- | --- | --- | --- | --- | --- |
|  | **Amp** | | **Kana** | | **Genta** | | **Erythro** | | **Clinda** | | **Cipro** | | **Nal** | | **Norflo** | | **Tet** | |
| CB1:6 | 4 | S | 8 | S | 1 | S | 1 | S | 2 | R | 16 | R | >128 | R | 128 | R | 0.25 | S |
| CB1:14 | 8 | S | 4 | S | 0.5 | S | 2 | S | 1 | R | 16 | R | >128 | R | 128 | R | 0.125 | S |
| CB1:18 | 8 | S | 8 | S | 1 | S | 2 | S | 0.5 | S | 8 | R | >128 | R | 64 | R | 0.25 | S |
| CB2:6 | 16 | R | >128 | R | 1 | S | 2 | S | 1 | R | 16 | R | >128 | R | 128 | R | 64 | R |
| CB2:8 | 16 | R | >128 | R | 0.5 | S | 2 | S | 0.5 | S | 16 | R | >128 | R | 128 | R | 64 | R |
| CB2:11 | 16 | R | >128 | R | 1 | S | 2 | S | 1 | R | 16 | R | >128 | R | 128 | R | 64 | R |
| CB3:1 | 8 | S | 8 | S | 1 | S | 4 | S | 2 | R | 0.5 | S | 16 | S | 1 | S | 64 | R |
| CB3:5 | 8 | S | 8 | S | 1 | S | 4 | S | 2 | R | 0.5 | S | 16 | S | 1 | S | 1 | S |
| CB4:21 | 4 | S | 8 | S | 0.5 | S | 2 | S | 1 | R | 0.25 | S | 4 | S | 2 | S | 32 | R |
| CB4:22 | 4 | S | 8 | S | 1 | S | 2 | S | 1 | R | 0.25 | S | 4 | S | 2 | S | 32 | R |
| CB6:9 | 8 | S | 8 | S | 0.5 | S | 1 | S | 1 | R | 0.25 | S | 4 | S | 0.5 | S | 64 | R |
| CB6:26 | 4 | S | 8 | S | 0.5 | S | 1 | S | 2 | R | 0.25 | S | 4 | S | 0.25 | S | 16 | R |
| CB7:15 | 4 | S | 8 | S | 1 | S | 1 | S | 1 | R | 16 | R | >125 | R | 64 | R | 128 | R |
| CB7:21 | 4 | S | 8 | S | 1 | S | 1 | S | 1 | R | 8 | R | >128 | R | 64 | R | 128 | R |
| CB8:14 | 4 | S | 8 | S | 1 | S | 2 | S | 2 | R | 1 | S | 4 | S | 0.5 | S | 32 | R |
| CB8:15 | 16 | I | 8 | S | 0.5 | S | 2 | S | 2 | R | 0.5 | S | 8 | S | 1 | S | 32 | R |
| CT1:1 | 32 | R | 4 | S | 0.5 | S | 4 | S | 1 | R | 32 | R | >128 | R | 128 | R | 128 | R |
| CT1:9 | 32 | R | 4 | S | 0.5 | S | 4 | S | 1 | R | 32 | R | >128 | R | 128 | R | >128 | R |
| CT2:2 | 8 | S | >128 | R | 1 | S | >128 | R | 32 | R | 32 | R | >128 | R | 64 | R | 128 | R |
| CT3:5 | 32 | R | >128 | R | 0.5 | S | >128 | R | 2 | R | 16 | R | 64 | R | 128 | R | 128 | R |
| CT3:19 | 16 | I | >128 | R | 1 | S | >128 | R | 8 | R | 32 | R | 128 | R | 128 | R | 128 | R |
| CT4:4 | 16 | I | >128 | R | 1 | S | >128 | R | 32 | R | 16 | R | 128 | R | 128 | R | 128 | R |
| CT4:14 | 16 | I | >128 | R | 1 | S | >128 | R | 32 | R | 16 | R | 128 | R | 128 | R | 128 | R |
| CT5:2 | 8 | S | >128 | R | 1 | S | >128 | R | 32 | R | 16 | R | 128 | R | 128 | R | 128 | R |
| CT5:8 | 8 | S | >128 | R | 1 | S | >128 | R | 32 | R | 16 | R | >128 | R | 128 | R | 128 | R |
| CT5:10 | 8 | S | >128 | R | 1 | S | >128 | R | 32 | R | 32 | R | 128 | R | 128 | R | 128 | R |
| CT5:12 | 8 | S | >128 | R | 1 | S | >128 | R | 32 | R | 32 | R | 128 | R | 128 | R | 128 | R |
| CT5:18 | 32 | R | >128 | R | 0.5 | S | >128 | R | 32 | R | 16 | R | 128 | R | 64 | R | 64 | R |
| CT6:8 | 8 | S | 8 | S | 1 | S | 1 | S | 1 | R | 1 | S | 16 | S | 0.25 | S | >128 | R |
| CT6:16 | 128 | R | 8 | S | 1 | S | >128 | R | 32 | R | 1 | S | 16 | S | 1 | S | >128 | R |
| CT6:18 | 16 | I | >128 | R | 1 | S | >128 | R | 8 | R | 16 | R | >128 | R | 64 | R | 128 | R |
| CT8:22 | 16 | I | 8 | S | 0.5 | S | 2 | S | 1 | R | 32 | R | >128 | R | 128 | R | 128 | R |
| CT8:28 | 16 | I | 8 | S | 0.25 | S | 1 | S | 1 | R | 16 | R | >128 | R | 128 | R | 128 | R |
| CT8:29 | 16 | I | >128 | R | 1 | S | 128 | R | 1 | R | 16 | R | >128 | R | 128 | R | 128 | R |
| CT9:14 | 8 | S | >128 | R | 0.5 | S | 4 | S | 1 | R | 32 | R | >128 | R | 128 | R | >128 | R |
| CT9:21 | 8 | S | >128 | R | 0.5 | S | 8 | R | 1 | R | 0.5 | S | 8 | S | 1 | S | >128 | R |
| CT10:18 | 4 | S | >128 | R | 0.5 | S | 2 | S | 0.25 | S | 0.5 | S | 4 | S | 0.5 | S | 128 | R |

^a^ S, sensitive; R, resistant; according to the clinical resistance cut-offs reported in the guidelines of the European Committee on Antimicrobial Susceptibility Testing (EUCAST, 2018)

Amp, ampicillin; Kana, kanamycin; Genta, gentamicin; Erythro, erythromycin; Clinda, clindamycin; Cipro, ciprofloxacin; Nal, nalidixic acid; Norflo, norfloxacin; Tetra, tetracycline
