# Supplementary material for: (-)-α-Pinene reduces quorum sensing and Campylobacter jejuni colonization in broiler chickens
Source: PLoS One. 2020 Apr 1;15(4):e0230423. doi: 10.1371/journal.pone.0230423 (PMC7112227; doi:10.1371/journal.pone.0230423)
Supplement: Attachment — Submitted filename: Simunovicetal2019_Response to Reviewers.docx [file pone.0230423.s005.docx]

**Response to reviewers**

Title of manuscript: (-)-α-Pinene reduces quorum sensing and *Campylobacter jejuni* colonization in broiler chickens

**Reviewer #1**

**1. The paper is largely based on a previous study of the same research groups (Kovac et al. 2015) in which both the antimicrobial activity and the resistance modulatory activities of (-)-α-pinene in C. jejuni against two fluoroquinolones was investigated. In the present paper, the C. jejuni strain collection was extended to confirm these activities, but surprisingly the strain (NCTC 11168) used for inoculation of the chickens was not included.**

**Answer:** The strain used for the chicken inoculation was also used in the *in-vitro* resistance modulatory part of the study. In Table 1, the last strain under the designation “11168” is the inoculation strain. We have changed this designation to “NCTC 11168” now, to avoid further confusion.

**2. In addition, now also the inhibition of quorum sensing was shown in a Vibrio harveyi model. Based on these observations, a broiler chicken infection model was used to see if (-)-α-pinene added in the drinking water would reduce the C. jejuni load in the gut. In contrast to the in vitro results, no resistance modulatory effect was seen in the chickens, but where are the in vitro data which proof this effect for the inoculation strain ?**

**Answer**: Please note that in Table 1, where the *in-vitro* resistance modulatory data are shown, the data for the inoculation strain (NCTC 11168) are shown in the bottom part. The resistance against ciprofloxacin showed a 2-fold change, and the resistance against erythromycin showed a 4-fold change.

**3. No effect, either (-)-α-pinene alone or in combination with enrofloxacin, was seen on the colonization level. Only through a direct gavage of (-)-α-pinene, a statistically significant but very modest reduction (< 1 log cfu/g feces) was observed for the ciprofloxacin sensitive C. jejuni population only. In fact, for half of the chickens infected with this sensitive C. jejuni population, the *C. jejuni* excretion level was comparable with the untreated control. The statistical significance was probably only obtained through one bird showing a considerable lower *C. jejuni* level. I therefore doubt about the soundness of the statistical analysis using a t-test and the inclusion of the outlier bird.**

**Answer**: As the data has a normal distribution, which was tested with Kolmogorov-Smirnov and Shapiro-Wilk tests (see Table1), students t-test is the appropriate statistical test here. We have now compared the statistical significance of the data with all of the data pointes (Table 2) and without the outlier (Table3, and the statistical significance, albeit weaker, still remains.

Table 1. Normality tests.

| **Tests of Normality** | | | | | | | |
| --- | --- | --- | --- | --- | --- | --- | --- |
|  | Group | Kolmogorov-Smirnov^a^ | | | Shapiro-Wilk | | |
|  |  | Statistic | df | Sig. | Statistic | df | Sig. |
| CjejuniNo | cipS | 0.228 | 10 | 0.151 | 0.900 | 10 | 0.221 |
|  | cipS-AP | 0.156 | 10 | 0.200 | 0.947 | 10 | 0.628 |
|  | cipR | 0.179 | 10 | 0.200 | 0.954 | 10 | 0.715 |
|  | cipR-AP | 0.145 | 10 | 0.200 | 0.952 | 10 | 0.687 |

Table 2. T-tests with all of the data.

|  | Levene's Test for Equality of Variances | | t-test for Equality of Means | | | | | | | |
| --- | --- | --- | --- | --- | --- | --- | --- | --- | --- | --- |
| CjejuniNo | F | Sig. | t | df | Sig. (2-tailed) | Mean Difference | Std. Error Difference | 95% Confidence Interval of the Difference | |  |
|  |  |  |  |  |  |  |  | Lower | Upper |  |
| Equal variances assumed | 1.287 | 0.271 | 2.397 | 18 | 0.028 | 0.7788 | 0.325 | 0.09613 | 1.462 |  |
| Equal variances not assumed |  |  | 2.397 | 14.26 | 0.031 | 0.7788 | 0.325 | 0.08307 | 1.475 |  |

Table 3. T-tests without outlier in the FQ-S (CipS) -AP group.

|  |  | Levene's Test for Equality of Variances | | t-test for Equality of Means | | | | | | |
| --- | --- | --- | --- | --- | --- | --- | --- | --- | --- | --- |
| Nooutlier |  | F | Sig. | t | df | Sig. (2-tailed) | Mean Difference | Std. Error Difference | 95% Confidence Interval of the Difference | |
|  |  |  |  |  |  |  |  |  | Lower | Upper |
| Equal variances assumed |  | 0.121 | 0.732 | 2.166 | 17 | 0.045 | 0.56984 | 0.26306 | 0.01483 | 1.12485 |
| Equal variances not assumed |  |  |  | 2.139 | 15.302 | 0.049 | 0.56984 | 0.26637 | 0.00305 | 1.13662 |

**4. Although the different categories of results are valuable on their own, there is a general lack of coherence between the results. The quorum sensing results have almost no connection with the other types of data and as they are only observed *in vitro,* there is no indication that this activity will also appear in vivo and therefore would help to find the mechanism of the observed *C. jejuni* colonization decrease with (-)-α-pinene direct gavage. “**

**Answe**r: The Introduction (L62-L63, L81-L88) has been changed and hopefully these changes will clarify and link the quorum sensing better with the rest of the data. We have explored quorum sensing inhibition as one of the possible mechanisms of (-)-α-pinene activity, together with the (previously published) efflux pump inhibition and membrane disruption. We are not proposing that quorum sensing inhibition is solely responsible for *C. jejuni* reduction after treatment with (-)-α-pinene, but rather that it is one of the actions of (-)-α-pinene against *C. jejuni* (L436 –L444).

**5. The rationale to look for a synergy between (-)-α-pinene and enrofloxacin in broiler chickens is not clear. Enrofloxacin is used in human medicine, but is not used in broilers to prevent or combat a C. jejuni infection. The observation of the enrofloxacin/ciprofloxacin resistance modulation by (-)-α-pinene in vitro is interesting and could have practical considerations for human clinical use, but I doubt about the practical relevance in poultry rearing.**

**Answer**: (-)-α-pinene was shown to modulate *C. jejuni* resistance to fluoroquinolone antibiotics (Table 1) and was expected to affect the emergence of fluoroquinolone resistant *Campylobacter* during antibiotic treatment. Thus a rationale to examine a synergy between (-)-α-pinene and enrofloxacin was warranted. We have modified the manuscript to clarify this point. The chicken model is a well established *in vivo* system to study fluoroquinolone resistance development in *Campylobacter*, which also occurs in human patients treated with a fluoroquinolone antibiotic. Thus the finding is relevant to human clinical cases where fluoroquinolones are commonly used to treat campylobacteriosis. Additionally, enrofloxacin is still being used in poultry (and other animals) in certain parts of the world for treatment of bacterial infections, such as colibasillosis and *Mycoplasma* infections. Due to prevalence of *C. jejuni* in poultry intestinal tract as a commensal, the treatment of poultry with enrofloxacin will result in resistance development in *C. jejuni*, which can be subsequently transmitted to humans via the food chain. If (-)-α-pinene is found to be effective in inhibiting resistance development in *Campylobacter*, it may be used as adjunct therapy to control the development of antibiotic-resistant *Campylobacter* in different hosts. In this study we used only one strain for the in vivo experiment. Additional work needs to be done to examine the potential of (-)-α-pinene.

**6. The introduction is written too general about the control of C. jejuni in poultry and the increasing risk of AMR in this pathogen. As a result, the real focus of this paper which is the effect of (-)-α-pinene on C. jejuni is not well introduced. For example, quorum sensing which is one of the focus points, is only introduced with a small paragraph of 5 lines, but mentioning only one reference related to C. jejuni in a guinea pig abortion model which is not really relevant for poultry.**

**Answer**: Thank you for this observation. We have edited the Introduction (L81-L98) and hope that this clarifies better the background and purpose of this study.

**7. The discussion is written too much as a review of literature on the effect of other essential oils or plant components on pathogens and on the importance of improving the control of C. jejuni in poultry in general. It’s not enough focused on the explanation and discussion of the own results. For example, there is no hypothesis why (-)-α-pinene has no effect on the ciprofloxacin resistant C. jejuni population in the broiler infection model.**

**Answer**: We have changed some parts of the Discussion for clarification (L381-L383; 431-432: 439-440), although we feel that the comparison of pinene with essential oils and their constituents is necessary as pinene is the major constituent of many essential oils.

We have attempted to explain the lack of effect of pinene on FQ-R strains by the increased fitness of FQ-R strains observed by Luo et al. (2005) (L430-L436). We feel that more speculation about the reason for the weaker effect observed here would be unwise, as we do not have more information at this point.

**8. There are still some corrections in English to be made, this should be checked throughout the manuscript.**

**Answe**r: The manuscript has been proofread and edited by a professional mother-tongue scientific editor.

**9. Other specific remarks:**

**-L145: for what purpose was the NCTC 11168 luxS mutant used? As negative control?**

**Answer**: Yes, it was used as a negative control. This has now been corrected in the manuscript.

**-L156: explain AB**

**Answer**: AB medium is described in the Bacterial strains and growth conditions (L111-L114).

**-L160: is the negative control correct here (V. harveyi suspension)?**

**Answer**: No, this was an error, and we apologize for it. It has now been removed from the manuscript.

**-L166 & 172: incorrect numbering of figures (Fig. S2 before S1)**

**Answer**: Thank you for this observation. The labeling has been corrected in text, and the Figure labels have remained the same.

**-L175-176: there can’t be two negative controls, the 11168 luxS mutant and fresh MHB?**

**Answer**: This is correct, and the error has been corrected. MHB is the “blank” value.

**-L191: explain MHA-SS?**

**Answer**: MHA-SS is described in Bacterial strains and growth conditions: L113-L115.

**-details for cloacal swabs**

**Answer:** We considered approximately 100 mg of feces per cloacal swab, and we have presented the data accordingly.

This is the method previously used in other studies (e.g., Sahin et al. (2003) **doi:** 10.1128/AEM.69.9.5372-5379.2003, and Luo et al. (2003) doi:[10.1128/AAC.47.1.390-394.2003](https://dx.doi.org/10.1128%2FAAC.47.1.390-394.2003)), and to maintain consistency and for better comparisons of our data with already published data, we would like to keep this as it is. The description has been added into the manuscript for clarification (L215-L220). Cloacal swabs are commonly used to detect *Campylobacter* shedding in poultry in a non-invasive way, as was used in the present study.

**-Campylobacter status of chickens was only checked by direct plating, not by enrichment? What was the detection limit**

**Answer:** Direct plating is considered a better option for *Campylobacter* spp. isolation from chicken feces, compared to enrichment (Musgrove et al., 2001; Sahin et al., 2003). The detection limit was 100 CFU/g feces. The description of the method has been adjusted for better clarification (L215-L220).

Musgrove, M.T., M.E. Berrang, J.A. Byrd, N.J. Stern, and N.A. Cox. 2001. Detection of Campylobacter spp. in ceca and crops with and without enrichment. Poult Sci. 80:825–828.

Sahin, O., Q. Zhang, and T.Y. Morishita. 2003. Detection of Campylobacter. In: Microbial Food Safety in Animal Agriculture. M.E. Torrence and R.E. Isaacson, eds. Iowa State Press, Ames, IA.183–193

**-L218: plated and incubated**

**Answer**: The text has been added to L218-L219.

**-L258, table 1: host origin of the strains can be shown here**

**Answer:** The description has been added as suggested.

**-L275: strains vs. isolates**

**Answer:** Corrected.

**-L280: also strain W14861 has this behavior**

**Answer:** Thank you for the observation. This has been corrected (L285).

**-L311-312: I don’t understand how the results are presented here versus the negative control (luxS mutant)**

**Answer:** The error was corrected for L314-317.

**-L324-328: this is a repetition from M&M**

**Answer:** Corrected.

**-L341: where are the horizontal lines in the figure?**

**Answer:** In each stack of data points there is a (small) horizontal line that represents the means of each dataset. Due to some technical difficulties with the Figure format, this was not presented properly. This has now been corrected.

**-L346: in Fig. 2, I only see 3 colonized birds**

**Answer**: Four birds are colonized, but the symbols are overlapping. The symbol with the thicker edges (the lowest) is actually two symbols. This could not be corrected on this graph.

**-L349: idem, only 7 colonized birds?**

**Answer**: As in the previous point, here we have two symbols on the upper part that are overlapping, and thus look like one symbol with thicker edges.

**-L366: what is the exact meaning of “the potential to lower…”, this is scientifically not well formulated; is it statistically significant or only a trend?**

**Answer**: This has been corrected.

**-L373-385: this is a repetition from M&M**

**Answer**: Corrected.

**-L439: macrolides were not included here**

**Answer:** Corrected. The sentence has been rearranged for clarification.

**Reviewer #2**

**1. The subject of the work is interesting and innovative. It raises an important issue regarding the search for alternative methods to combat Campylobacter in poultry. It would also be worth checking (maby in next stage of research) what effect the (-)-a-pinene has on other microorganisms inhabiting the intestines in birds. Does it not inhibit the growth and colonization of beneficial microflora such as lactobacilli or bifidocabterie?**

**Answer:** Thank you for this observation. Any treatment added to chicken water that can affect *C. jejuni* numbers will probably also influence other members of the intestinal microbiota, although it does not necessarily mean a negative influence on positive microbes, as are bifidobacteria. This is something that will indeed be considered for future work.

**2. I have some reservations about the methodology of the experiment regarding the assessment of the influence of a-pinene on the colonization of Camylobacter in chicks. The authors took cloacal swabs, diluted in MHB and plated on agar; the result is given in cfu/g feces. How did the authors weigh droppings when they took a swab?**

**Answer:** Each swab diluted was considered to have approximately 100 mg of fecal matter and was diluted in 1 mL of MHB so as to get a 10-fold dilution. Dilutions were plated and data are shown as cfu/g feces, with a detection limit of 100 cfu/g feces. We considered 100 mg of feces per cloacal swab and presented the data accordingly.

This is the method previously used in other studies (e.g., Sahin et al. (2003) **doi:**10.1128/AEM.69.9.5372-5379.2003 and Luo et al. (2003) doi:10.1128/AAC.47.1.390-394.2003, and for consistency and better comparisons of our data with already published data we prefer to keep this as it is. A description has been added into the manuscript for clarification (L209-L212). Cloacal swabs are commonly used to detect *Campylobacter* shedding in poultry in a non-invasive way, and it was used in the present study as such.

**3. The authors also tend to repeat in the Results chapter information from the Materials and Methods chapter, e.g. P17L324-328; P19L373-377.**

**Answer:** Corrections have been made to these parts of the text.

**Editor**

**As is often the case, the two reviewers have highlighted different points in their reviews.  I would urge the authors to read these comments carefully for any revision they make, as there are quite a few of them.  However, in terms of what needs to be addressed, the following four points can be made:**

1) **Methodological issues - sampling as swabs, but reporting data as if it were faeces, and concerns over the impact of outlier data in the conclusions drawn**

**Answer:** Corrections and additional explanations have been added into the text to explain the swabbing process better.

Additional statistical analysis was performed using all of the data and the data without the outlier, and the statistical significance remained. So we believe that the effect of pinene shows real significance.

**2)    Repetition of methods and results - both reviewers comment on this**

**Answer:** This has been corrected.

**3)    Justification for issues around the study design - relevance of the study to humans in terms of drug use in humans and chickens**

**Answer:** Corrections have been made to the text to clarify this matter. We do not propose that enrofloxacin should be used for *Campylobacter* control in poultry, and we apologize for the confusion.

**4)    Discussion - some clarification over aspects in regards to framing the results in the current literature**

**Answer:** Some modifications have been made throughout the Discussion (L395-L398; 435-443: 486-488).

**Journal requirements**

**1. When submitting your revision, we need you to address these additional requirements.**

**Please ensure that your manuscript meets PLOS ONE's style requirements, including those for file naming. The PLOS ONE style templates can be found at** [**http://www.journals.plos.org/plosone/s/file?id=wjVg/PLOSOne_formatting_sample_main_body.pdf**](https://nam01.safelinks.protection.outlook.com/?url=http%3A%2F%2Fwww.journals.plos.org%2Fplosone%2Fs%2Ffile%3Fid%3DwjVg%2FPLOSOne_formatting_sample_main_body.pdf&data=02%7C01%7Cjzk303%40psu.edu%7C50ccfe8c530e45f3a77a08d75550bb31%7C7cf48d453ddb4389a9c1c115526eb52e%7C0%7C1%7C637071677561043197&sdata=d7Bi8iEn7OTPYD6E8svNxaUKs61S%2FFa6QQFAT9CdqfI%3D&reserved=0) **and** [**http://www.journals.plos.org/plosone/s/file?id=ba62/PLOSOne_formatting_sample_title_authors_affiliations.pdf**](https://nam01.safelinks.protection.outlook.com/?url=http%3A%2F%2Fwww.journals.plos.org%2Fplosone%2Fs%2Ffile%3Fid%3Dba62%2FPLOSOne_formatting_sample_title_authors_affiliations.pdf&data=02%7C01%7Cjzk303%40psu.edu%7C50ccfe8c530e45f3a77a08d75550bb31%7C7cf48d453ddb4389a9c1c115526eb52e%7C0%7C1%7C637071677561043197&sdata=I59c0MpM39tPUwtSqIywbTU2UmfFq0xA64UY80xKI0k%3D&reserved=0)

**Answer:** Corrections have been made throughout the manuscript.

**2. In your Methods section, please provide additional details regarding the animals used in your study and ensure you have described the source. For more information regarding PLOS' policy on materials sharing and reporting, see** [**https://journals.plos.org/plosone/s/materials-and-software-sharing#loc-sharing-materials**](https://nam01.safelinks.protection.outlook.com/?url=https%3A%2F%2Fjournals.plos.org%2Fplosone%2Fs%2Fmaterials-and-software-sharing%23loc-sharing-materials&data=02%7C01%7Cjzk303%40psu.edu%7C50ccfe8c530e45f3a77a08d75550bb31%7C7cf48d453ddb4389a9c1c115526eb52e%7C0%7C1%7C637071677561053193&sdata=AtNcgxtsxLajjBlECSwcp1c1KWnUItksOPHk73TCH6k%3D&reserved=0).

**Answer:** Additional details in the method section have been added (L184-L185).

**3. We note that you have stated that you will provide repository information for your data at acceptance. Should your manuscript be accepted for publication, we will hold it until you provide the relevant accession numbers or DOIs necessary to access your data. If you wish to make changes to your Data Availability statement, please describe these changes in your cover letter and we will update your Data Availability statement to reflect the information you provide.**

**Answer:** DOIs for all of the data are listed in the data availability statement together with links that reviewers can use to access the data before it is published. DOIs will be activated upon manuscript acceptance. Appropriate DOIs (but still inactive) are also listed below.

**Data availability:**

Figure 1.

Doi: 10.6084/m9.figshare.8234153

Figure 2.

Doi: 10.6084/m9.figshare.8234192

Figure 3.

Doi: 10.6084/m9.figshare.8234189

Figures S1 and S2

Doi: 10.6084/m9.figshare.8234156

Tables S1 and S2.

Doi: 10.6084/m9.figshare.8234159
